# Morphological and Surgical Overview of Adolescent Testis Affected by Varicocele

**DOI:** 10.1155/2013/469413

**Published:** 2013-11-20

**Authors:** Giuseppe Santoro, Carmelo Romeo

**Affiliations:** ^1^Department of Biomorphology and Biotechnologies, A.O.U. “G. Martino”, University of Messina, Via Consolare Valeria, 98125 Messina, Italy; ^2^Department of Pediatric, Gynaecological, Microbiological and Biomedical Sciences, A.O.U. “G. Martino”, University of Messina, Via Consolare Valeria, 98125 Messina, Italy

## Abstract

Varicocele is a common pathology of the testis frequently associated with infertility. For its management, a fine morphological study of the testis, both macroscopically and microscopically, and an accurate choice of surgical procedure are mandatory. The present review focuses its attention on the anatomic substrates of adolescent varicocele and its pathophysiologic modifications. The comprehensive assessment of all the reported alterations should be considered by the clinician before deciding the type of treatment and the timing.

## 1. Introduction

The study of history of medicine has always shown a tight relationship between anatomy and surgery. If anatomy, in the ancient times, has provided knowledge for operative surgery thanks to the scalpel, in the modern times, the advent of instrumental investigations, both macroscopically and microscopically, helps surgeons define a full diagnosis of different pathologies and choose a proper surgical procedure with reduced surgical time and hospital length of stay and good cosmetic results. The present review shows the synergy between anatomy and surgery in the management of adolescent varicocele.

Varicocele is a common pathology of the testis characterized by a varicosity of the pampiniform venus plexus of the spermatic cord, with an incidence in older adolescent of 12,4–17,8%, with an average of 14,7% [1]. Once a varicocele has developed, it persists in adulthood [2]. Less frequently, bilateral varicocele is present. Right-sided only varicocele is exceedingly rare and should advice a prompt investigation for a retroperitoneal mass that could compromise the venous return from the right testicle [3]. There is a considerable debate about the effects of varicocele on future fertility, but the current evidence suggests that varicocele is found in a higher percentage among males attending the infertility clinics and that treatment of varicocele is associated with increased spontaneous conception rates among infertile couples [4].

## 2. Aetiology

Considering the predominance of the left side varicocele and the unique anatomy of the left testicular vein (LTV), many theories were postulated to explain the aetiology of varicocele: incompetence of the venous valve system [5], hydrostatic pressure difference between the longer LTV [6], nutcracker effect [7, 8], and increased arterial blood flow to the testis at puberty that exceeds the venous capacity resulting in venous dilatation [9, 10].

## 3. Pathogenesis

The pathogenesis of testicular damage remains also controversial and many mechanisms have been proposed, including induced testicular hypoxia by venous stasis and small vessel occlusion, leading to Leydig cell and germinal cell dysfunction [11], retrograde blood flow of adrenal and renal metabolites in the spermatic vein [12], increased testicular and scrotal temperature [13], reduction of gonadotrophin and androgen secretion [14], alterations in the axis lamina propria-extracellular matrix- (ECM-) germinal epithelium [15], and increased nitric oxide (NO) production within dilated spermatic vein [16].

## 4. Gross Anatomy

The testes are a pair of oval, slightly flattened bodies of whitish colour, measuring about 4 cm in length, 2–5 cm from before backwards, and rather less in thickness. Each testis is placed within the cavity of the scrotum in such a manner that its long axis is directed upwards and slightly forwards and laterally. Usually the left gland is a little lower than the right one [17].

The testis is supplied by the testicular artery, a branch of the abdominal aorta. It is a slender vessel which, after a long course from the abdomen to the scrotum, reaches the posterior border of the testis, where it breaks up into branches [17]. It has been observed that there are commonly two main branches which pass forwards, one on each side of the testis, to ramify on the deep surface of the tunica albuginea in the tunica vasculosa [18]. From this vascular plexus the small terminal arteries pass backwards into the substance of the organ along the septula and converge on the mediastinum testis [18]. The arterial blood supply of the testis comes also from the artery of the ductus deferens and the cremasteric artery, which anastomose with the testicular artery [19].

**Figure 1:**
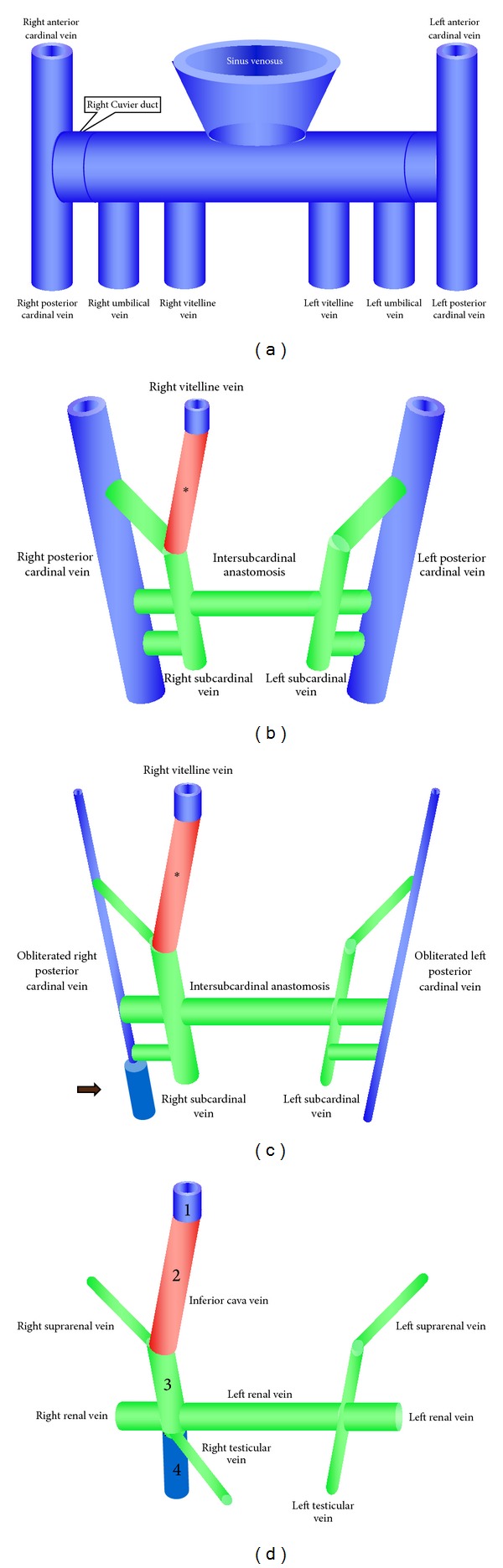
(a) Symmetric stage of the human vascular system development (4-week embryo). Three symmetric paired veins form the basis of the early venous system, draining into the heart: vitelline veins, umbilical veins, and cardinal veins. Both cardinal veins empty into the common duct of Cuvier. (b) Asymmetric stage of the human vascular system development (5-week embryo). Note the persistence of only the right vitelline vein, which, together with the hepatic segment, forms the upper portion of the inferior vena cava (∗). The posterior cardinal veins appear as paired, symmetrical vessels extending from the duct of Cuvier to the tail region. In the subhepatic region, paired vessels (right and left subcardinal veins) and intersubcardinal anastomosis are formed. (c) At the 6th week posterior cardinal veins are completely obliterated on the left side, whilst, on the right side, only the inferior part remains (arrow). A similar process involves the left subcardinal vein. On the right side, the subcardinal vein is maintained, thus forming the intermediate part of the inferior vena cava. (d) At the end of the developmental processes the right testicular vein drains into the definitive inferior vena cava, formed by four parts (1, 2, 3, and 4) of different origins. The left testicular vein drains in the left renal vein, derived from the intersubcardinal anastomosis.

The veins running along the septula and those of the tunica vasculosa converge on the posterior border of the testis where they form a dense plexus, called the pampiniform plexus (PP), which finally pours its blood through the TV, on the right side, into the inferior vena cava (IVC), on the left side, and into the left renal vein (LRV) [17]. The origin of the different venous drainage of right and left TVs is not fully understood. It is tightly connected to the ontogeny of the IVC. During the vascular system development, three symmetric paired veins form the basis of the early venous system in the 4-week human embryo (symmetric stage), draining into the heart: vitelline veins, umbilical veins, and cardinal veins (CVs) [20–22] (Figure 1(a)). The CVs drain the embryo body, with the anterior and posterior ones draining the cranial and caudal parts of the body, respectively. Both CVs empty into the common duct of Cuvier (DC), which represents the third venous system entering the venous sinus of the embryonic heart [20–22]. The posterior cardinal veins appear early as paired, symmetrical vessels, extending from the DC to the tail region, and are placed ventrolaterally to the aorta [22] (Figure 1(b)). While the anterior CVs persist for their most part as permanent vessels, the posterior ones undergo from the 5th week of development (asymmetric stage) regressive changes and new bilateral vessels take place, in particular the subcardinal veins (Figure 1(b)). At the 6th week of development, numerous intersubcardinal anastomoses are formed (Figure 1(b)). Subsequently, the obliteration of the left branches, which are connected to the right ones, leads to the development of the intermediate part of IVC [20–22] (Figure 1(c)). Since on the left side the original anastomosis between the subcardinal and cardinal is maintained as the left renal vein (LRV), the LTV is a branch of the LRV draining as a remnant of the left subcardinal veins. The right testicular vein (RTV) may be considered as branch of the IVC which is independent of the cardinal vein [22] (Figure 1(d)). In fact in a man with two IVC, the RTV drained into the confluence of the right IVC with the ipsilateral RV, while the LTV drained into the LRV in spite of the presence of the left IVC [23]. Venographic studies have previously demonstrated that the LTV can rarely enter the IVC, and communications exist between the testicular vein and the IVC below the level of the RVs [24–26]. Moreover it has been observed that the PP can also drain via pudendal veins and cremasteric veins [27, 28] and cross-communications between the left and right testicular venous systems were also demonstrated [5, 6, 25, 29]. Finally, it is important to note that, swing to their different origins, the RTV drains obliquely into the IVC, while the LTV drains at a right angle into the LRV. In addition, the insertion of the LTV is 8–10 cm higher than that of the RTV which results in 8–10 cm greater pressure on the blood flow from the LTV that will lead to varicocele formation. Similarly, considering that the TVs contain valves which help prevent retrograde flow of blood, absent or defective valves will subsequently lead to an increase in the pressure within the spermatic veins [4], as it happens for the compression of the LRV between the aorta and the mesenteric superior artery (nutcracker phenomenon) [30–33].

The lymph vessels of the testis pass upwards in the spermatic cord and end in the lymph nodes at the sides of the aorta and inferior vena cava below the renal veins [17].

The nerves for the testis and epididymis accompany the artery and are derived through the aortic and renal plexuses from the tenth thoracic segment of the spinal medulla [17].

## 5. Microscopic Investigation (Figure 2)

Another morphological remark is the microscopic investigation of the testicular parenchyma based on the numerous histological alterations observed in both intra- and extratubular compartments of varicocele testis. The human seminiferous tubules are formed by a very complex stratified epithelium containing spermatogenic cells in different stages of development and supporting cells, named the Sertoli cells (SC). In the angular interstices between various seminiferous tubules, there are Leydig cells (LC), which represent the endocrine component of the testis [15]. Both SC and LC regulate spermatogenesis by steroidogenesis and growth factors production [1]. In varicocele patients the intratubular compartment presents a wide array of abnormalities: Leydig cells hyperplasia, decreased number of spermatogonia per tubule, spermatogenesis arrest, and sloughing of germinal epithelium [1]. The intratubular compartment is normally surrounded by a lamina propria composed of a double-layered basal lamina and 5–7 external cellular layers [34]. The peritubular basal lamina, composed of extracellular matrix (ECM) components, is formed by two thin layers: the inner electron-transparent layer, situated close to the plasma membrane of the germinal epithelium, the lamina lucida or rara, and the outer electron-dense layer, in close relationship to the first extracellular layer of the lamina propria, the lamina densa [35]. In adolescent varicocele testis, the peritubular basal lamina shows an uneven profile with a variable thickness and it is altered in two of its major ECM components: laminin and collagen type IV [36]. These two molecules, apart from a mechanical linkage, play a key role in the regulation of major biological cellular functions. Through the relationship with the adhesion receptors of the integrin family localized in the cellular membrane and consequently with the intracellular actin-associated proteins, messages are transmitted from the ECM to the nuclear compartment thus controlling the ubiquitous process of differentiation, proliferation, adhesion, migration, gene expression, and spermatogenesis. For this reason it has been hypothesized that the observed modifications of morphology and composition of the peritubular basal lamina could represent a mechanism responsible for the lesions characteristic of varicocele [15]. To support this hypothesis two actin-associated proteins of the adherens junctions, talin and vinulin, expressed in the testis, have been investigated in adolescent with varicocele. Particularly, in normal adolescent testis in Sertoli and Leydig cells vinculin is expressed at the cell-cell and cell-ECM adherence junctions, while talin is present only at the cell-ECM adherence junctions level. In adolescent affected by varicocele, there were slight testicular alterations of vinculin and talin only in Sertoli cells, while the Leydig cells present an expression and distribution similar to the normal testis. Sometimes, in the same sample, altered seminiferous tubules were found adjacent to normal ones. These pieces of evidence were considered a possible not well-consolidated and -generalized tubular damage [37]. On the basis of these results, it was speculated that the modifications in two components of the adherens junction, that is, basal lamina and actin-associated proteins, could negatively influence the spermatogenesis in adolescent varicocele testis [15, 37]. In this regard, an altered peritubular basal lamina and changes in Sertoli cell junctions in tubular regions where germ cells were depleted have been reported [38]. The lamina propria of normal adolescent seminiferous tubules is also composed of 3–5 layers of myofibroblasts and 1 or more outer layers of fibroblasts; these cells are submerged in a framework of extracellular glycosaminoglycans (GAGs), proteoglycans, and collagen fibers. The myofibroblasts are individual flat cells, with a diameter of 40–60 *μ*m, and their cytoplasm split up into two or more layers; they do not form continuous cell layers and are not completely covered by a basal lamina [34, 39]. They are responsible for the contraction of the seminiferous tubules necessary for the transport of testicular spermatozoa and fluid and take part in the regulation of spermatogenesis and the creation of blood testis barrier [40, 41]. In adolescent affected by varicocele the myofibroblasts still maintain their morphological features with only mild alterations [39]. Moreover they do not transform into fibroblasts [39], as it is already demonstrated, in adult varicocele, that corresponds to the reported progressive sclerosis of the lamina propria [42]. Their integrity may be an important feature of adolescent varicocele [15]. Finally, the lamina propria shows a maximum thickness of 10 *μ*m and a surface area between 3970 and 4472 *μ*m^2^, while the tubular diameter is between 158 and 200 *μ*m^2^. In adolescent affected by varicocele, the lamina propria shows different degrees of thickening till a maximum value of 35 *μ*m. This is the result of an increased deposition of extracellular components, starting from the innermost layer of collagen fibers extending to the outer extracellular layer [34]. This condition is responsible for the formation of deep invaginations facing the germinal epithelium, which, in other testicular pathologies, is ascribed to tubular damage caused by a blockage in the mediation of the lamina propria between the interstitium and the germinal epithelium [43]. The increased thickness of the lamina propria corresponds to a progressive increase in its surface area and a reduction in the tubular diameter [34]. 

Aquaporins (AQP) are membrane channel proteins that facilitate rapid passive movement of water. They have been identified in different tissues and also in the testis. In particular in varicocele testes AQP-1 displays an overexpression at venular endothelial cell membranes together with a previously unreported positive expression at the cell membranes of the Sertoli cells, immature germ cells, and Leydig cells. The overexpression could suggest an involvement of such a protein in trying to balance the excessive endotubular and extratubular fluid excess due to varicocele [44]. Moreover AQP-9 expression has also been investigated in normal and varicocele human testis. AQP-9 seems to have a key role in the water and lactate transport from Sertoli cells to germ cells. AQP-9 appeared to be focal or lacking in adolescent varicocele testes, suggesting AQP-9 to be downregulated in such testicular disorder. It is then possible to speculate that this reduced expression could lead to lactate deprivation in germ cells with subsequent hypospermatogenesis [45]. 

In the last years great importance has been given to the role played by NO in the regulation of male reproductive function and fertility, Leydig cell function, myofibroblast contraction, and hence tubular peristalsis; on the contrary its over production can be harmful for both testicular and sperm functions [15]. In adolescent affected by varicocele NO overproduction is present within dilated spermatic veins independent of age or time of onset of symptoms [46]. The origin of NO hyperproduction has also been investigated. Leydig cells of adolescents constitutively express the inducible isoform of NO synthase (i-NOS). Under pathological conditions, such as varicocele, i-NOS is upregulated and is a possible source of NO overproduction [47]. It was hypothesized that in adolescent with varicocele a condition of oxidative stress is already present which does not seem to be correlated with the age of patients and/or the duration of symptoms and it is limited to the testis. This stress is due to the hyperproduction of NO and to its more reactive metabolites such as peroxynitrites. Effects can be at different levels: vasal level: NO determines vasodilatation and contributes to the status; myofibroblasts level: a prolonged condition of myorelaxation and consequently inhibition of tubular peristaltic activity is present; hormonal level: persisting the pathological condition of NO overproduction, the testosterone synthesis by Leydig cells could be altered; sperm level: excessive levels of free radicals can alter the normal physiological function of spermatozoa [16].

Another important morphological study for varicocele diagnosis should be the semen analysis characterized by increased number of pathological sperm forms, decreased motility, and decreased sperm density and motility [1]. Unfortunately semen analysis is mostly impractical in adolescent population and no established norms exist for adolescent semen analysis [1, 3, 48]. 

## 6. Varicocele Grading and Evaluation

Varicocele should be evaluated in the standing position, in a warm room. The scrotum should be first examined to rule out evident discrepancy in the aspect with evident dilatations of the veins of the pampiniform plexus. Afterward the patient should perform a Valsalva manoeuvre. With the patient supine the palpation of the testes should be performed to check for testis consistency and volume discrepancy. The evaluation of varicosity, according to the most used classification proposed by Dubin and Amelar, should be graded as follows: I = small sized only palpable during Valsalva manoeuvre, II = medium sized palpable at rest, and grade III = large sized visible at rest [49].

Secondary varicocele can be caused by serious conditions, like tumors, and should always be considered, especially if evident in the less common right side or if does not change, rapidly, its size from the upright to the supine position.

In addition, varicocele can be classified according to the degree of reflux identified by colour Doppler ultrasound scan: grade I = reflux induced by Valsalva manoeuvre with pattern 1, only very little reflux at the beginning of the Valsalva, or pattern 2, reflux during the full length of the Valsalva; grade II = intermittent spontaneous venous reflux, and grade III = continuous spontaneous venous reflux [50]. Doppler ultrasound should always be used to measure the testis volume, compare discrepancy between the two testes, if present, and to measure the maximum vein diameter and the peak retrograde flow (PRF) in the supine position [51].

Venography can identify the enlargement of the pampiniform plexus with reflux of blood into its tributaries. In addition, it can identify collateral vessels and incompetent valves. However this method has widely been replaced with other tests that are less invasive, less time consuming and determine lower exposure to radiation [52].

Another fundamental morphological consideration for varicocele management is the evaluation of the testicular volume; in fact significant testicular volume loss is observed in varicocele testes. Testicular volume during preadolescents is constant and at the onset of puberty the testis suddenly increases in size even prior to other pubertal changes. In adolescents with a varicocele the rapid growth of the testis between the ages of 11 and 16 is effected by the varicocele and results in a volume discrepancy between the right testis and left testis [1]. Particularly it has been reported in 77% of boys, 10% of whom had a left testis one-fourth the size of the right testis [53]; moreover the testicular hypotrophy is time dependent [54, 55]. For Paduck the testicular growth arrest may be considered the hallmark of testicular damage in adolescent varicocele, and the decrease in testicular volume is the best indicator for surgical correction of a varicocele [1]. In adolescents with ipsilateral testicular hypotrophy, it has also been shown that catch-up growth can be readily achieved in 50% to 80% of patients after varicocelectomy [56–59]. The ipsilateral testicular hypotrophy has received numerous classifications: as a testicular volume differential of 2 mL or more [60, 61], a volume difference of 10% or greater, 15% or greater, and 20% or greater, and a volume difference of 2-3 cc between testes [56, 57, 62, 63]. Anyway Glassber affirmed that the most used formula for left asymmetry is as follows: ([right testis volume − left testis volume]/right testis volume) × 100; volumes are measured in cubic centimetres [64]. Salzhauer used a percentage differential when evaluating hypotrophy because a 2 mL differential between the much smaller testicles in early puberty will represent a much more significant difference than in late puberty, when the testicles are much larger [3]. 

## 7. Indication for Treatment in the Adolescent

On the basis of their previous research, Kass and Reitelman suggested treating varicocele if one of the following conditions is present: (1) semen analysis is abnormal; (2) left testicular volume is at least 3 mL less than that of the right one or a more than 10% reduction in volume; (3) the response of either luteinizing hormone or FSH to Gn-RH stimulation is supranormal; (4) there are palpable varicoceles bilaterally; or (5) there is a large symptomatic varicocele [65]. Nowadays all these parameters are considered indications for treatment of varicocele in the adolescent age except for the semen analysis since it is considered impractical to obtain a semen sample in this age group in most centers around the world. More recently spermatic venous diameter greater than 3 mm and PRF 30 cm per second or greater are considered helpful to identify patients at a higher risk for progressive testicular atrophy and then to follow up them more closely [51]. Moreover intense physical activity has to be considered as an aggravating factor in the natural history of varicocele [66]. In countries, such as Italy, in patients with clinical evident varicocele (grades II and III) and necessity of sport eligibility for agonistic sport practice, varicocele is usually at this stage an indication for treatment [66].

Aside from the debate related to what cutoff to be used, there is a debate regarding how long patients with asymmetry should be followed up before deciding on surgery [64]. Recently, a significantly higher incidence of patients with decreased sperm concentration and decreased total motile sperm in patients with a sonographically derived volume differential of as little as 10% has been reported [67]. Of the 57 boys in this study, those with volume differentials of 10–20% had an 11% chance of having a subnormal total motile sperm count. If the testicular differential exceeded 20%, the total motile sperm count was abnormal in 59%. Diamond recommended varicocelectomy when a greater than 20% asymmetry is identified and persists for more than 1 year, whereas Kolon suggested following patients with asymmetry even longer since catch-up growth was found in 71% of boys at a mean of 3 years without any intervening surgery [68].

These findings have led to the common usage of testicular size as a surrogate for potential fertility. It is generally accepted that a normal testis volume differential is less than 2 mL or 20% [69]. Current surgical indications for an adolescent varicocelectomy include a size differential greater than 20% (greater than 2 mL) between testicles or a testicular size decrease greater than 2 standard deviations from the normal growth curve [70].

## 8. Treatment Options

Different techniques have been proposed to treat varicocele in the adolescent age. These techniques can be divided into two different categories: (a) percutaneous embolization; (b) surgical procedures, open and laparoscopic. 

Percutaneous embolization can be performed both through a retrograde route via the femoral vein or an anterograde route via the incannulation of one of the dilated veins of the pampiniform plexus utilizing a small incision on the scrotum. These techniques can be performed as day case procedure with local anesthesia. The embolization can be performed with either a sclerosant agent or solid embolic devices under fluoroscopy.

The open surgical procedures are the most used techniques to repair varicocele in the adolescent age. There are two classical routes to ligate the spermatic veins: (i) abdominal incision, with en mass ligation of spermatic artery and vein (Palomo technique); this technique has been nowadays substitute by the Palomo laparoscopic ligation; (ii) inguinal incision, to ligate the spermatic vein(s) in the inguinal canal (Ivanissevich technique). More recently the so-called subinguinal varicocelectomy has gained popularity. With this technique the spermatic veins are ligated, usually using magnification device, and the spermatic artery and lymphatics are spared. The inguinal approaches can be performed either under local or general anesthesia. 

With the development of laparoscopic techniques also in the pediatric population in the last decade the laparoscopic ligation of the spermatic vein and artery has been proposed with success. This technique should be performed under general anesthesia.

The overall results of all the techniques described in adult have been recently reported in a meta-analysis. The higher recurrence rate is for the Palomo technique (14.9%), followed by the radiologic embolization (12.7%) and laparoscopic varicocelectomy (4.3%). The lowest recurrence rate is for the microsurgical inguinal technique (1.05%) followed by the inguinal Ivanissevich technique (2.63%). Hydrocele is usually a common complication particularly after the Palomo technique (8.24%), the Ivanissevich (7.3%), and the laparoscopic varicocelectomy (2.84%), but it is extremely rare after microsurgical varicocelectomy (0.44%). The overall spontaneous pregnancy rate is also significantly better after microsurgical varicocelectomy compared to other techniques [71].

A recent meta-analysis and literature review performed on eleven studies published between 2000 and 2009, with a population of 1443 children and adolescents treated, have compared the two most popular approaches in the pediatric age group reported in the literature: the laparoscopic and the open techniques [72]. Varicocele recurrence in adolescents who underwent laparoscopic versus open techniques has no statistical difference (4.7% versus 8.6%). Similarly no statistical difference could be demonstrated in terms of postoperative hydrocele formation: 9.5% for the laparoscopic technique versus 6.7% for the open techniques [72].

 Other interesting data derived from the above meta-analysis is that the injection of blue dye before laparoscopic ligation significantly reduces the incidence of hydrocele formation and that ligation of both artery and veins during laparoscopic varicocelectomy significantly reduces the recurrence rate [72].

There are very few reports on the effect of varicocele treatment on future fertility in the pediatric population. One recent paper has analyzed the long term (12–17 years) effects in terms of pregnancy rate with an overall success rate in 75% of treated patients with significant improvement in sperm count, abnormal forms, and viability compared to the preoperative examination [73].

## 9. Conclusions

The present review has focused its attention on the anatomic substrates of adolescent varicocele and its pathophysiologic modifications. Considering that morphological and biomolecular alterations are already recorded in adolescent varicocele and could interfere with cellular migration, differentiation, and nutrition, it is important to consider the correction of varicocele, aside from the classical indications, also as an option to arrest the progressive damage that inevitably threatens for several years the adolescent testis. This comprehensive assessment should be considered when the clinician must decide the right operation at the best time for the correct patient.

## Figures and Tables

**Figure 2:**
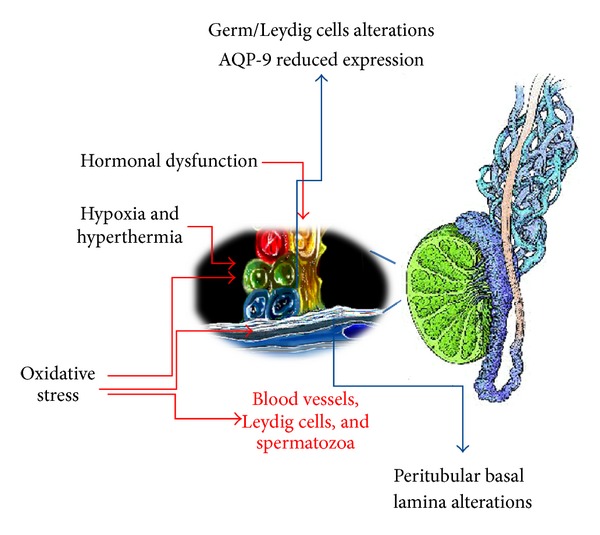
Different pathogenetic mechanisms of testis damage (red arrows) and different morphological alterations (blue arrows) observed in adolescent patients with varicocele. These mechanisms have been demonstrated in the adolescent age and could threaten testis throughout the growth.
